# Metagenomics Analysis Reveals Compositional and Functional Differences in the Gut Microbiota of Red Swamp Crayfish, *Procambarus clarkii*, Grown on Two Different Culture Environments

**DOI:** 10.3389/fmicb.2021.735190

**Published:** 2021-10-18

**Authors:** Xi Chen, Limin Fan, Liping Qiu, Xinxu Dong, Qing Wang, Gengdong Hu, Shunlong Meng, Dandan Li, Jiazhang Chen

**Affiliations:** ^1^Freshwater Fisheries Research Center, Chinese Academy of Fishery Sciences, Scientific Observing and Experimental Station of Fishery Resources and Environment in the Lower Reaches of the Yangtze River, Wuxi, China; ^2^Wuxi Fisheries College, Nanjing Agricultural University, Wuxi, China; ^3^Wuxi COFCO Engineering & Technology Co., Ltd., Wuxi, China

**Keywords:** metagenomics, rice-crayfish cultivation, gut microbiota, function, red swamp crayfish

## Abstract

The structure and function of intestinal microorganisms are closely related to host metabolism, development, physiology, and health. The red swamp crayfish, *Procambarus clarkii*, is an important farmed aquatic species in China, which is grown in aquaculture ponds and rice paddy fields. Since these are two distinct cultivation environments with important differences in nutrient input and ecological community composition, we hypothesized that they may have different effects on the gut microbiota of the crayfish. Here, we sought to examine this hypothesis. To that aim, metagenomics analyses were applied to unveil the taxonomic composition and functional diversity of the microbiota in the intestines of red swamp crayfish grown in aquaculture ponds and rice-crayfish cultivation environments. The results showed that Firmicutes and Proteobacteria were the two most abundant microbial components. In addition, the relative abundance of bacterial and archaeal communities, but not that of fungal and viral communities, significantly differed between the two environments. The abundance of genes involved in pathways related to genetic information processing and human diseases was lower in the guts of red swamp crayfish grown in rice-crayfish cultivation environments. In particular, the abundance of two gene sets, K13730 and K08303, which are related to epithelial cell invasion by *Listeria monocytogenes* and *Helicobacter pylori*, respectively, decreased in this culture environment. In addition, the samples from rice-crayfish cultivation environments tended to have lower relative abundance of glycosyltransferases (GTs), which were the most abundant carbohydrate-active enzymes in the samples from both groups, higher abundance of glycoside hydrolases, and lower abundance of GT2.

## Introduction

The red swamp crayfish, *Procambarus clarkii*, which is an invasive species in Europe and Asia, is also a highly adaptable aquaculture species that is gradually becoming an important aquatic food sector among fishery trading commodities in China (Du et al., 2017). The pond monoculture and rice-crayfish cultivation models are the two main farming models used for crayfish in China. Originally, crayfish were mainly cultured in ponds; however, the rice-crayfish cultivation model has developed rapidly in recent years. In 2019, red swamp crayfish farming in rice paddy fields accounted for 86% of the total production in China (Crayfish Industry Report 2020)^1^. The original model has been practiced in Louisiana, United States, for several decades (Chien and Avault, 1980; Johnson and Avault, 1982); however, the optimizations carried out for rice-crayfish cultivation helped the red swamp crayfish to become one of the most economically important farmed aquatic species in China (Shui et al., 2020).

The crayfish aquaculture pond and rice-crayfish cultivation ecosystems differ in many ways, including water depth, crayfish biomass, and planting of rice. Research (Guohan et al., 2017) has also indicated that rice-crayfish cultivation increases the microbial biomass in soil compared with that in the monoculture model. Thus, the differences in these two crayfish living environments might have different effects on the growth and/or physiological status of crayfish. Therefore, it is necessary to screen indicators and assess the significance of these influences.

The gut microbiota plays a critical role in host development, physiology, and health (Kristen et al., 2012). This is similar in aquatic animals (Gómez and Balcázar, 2010; Roeselers et al., 2011; Wang, 2018). Many factors have been reported to influence gut microbes, including biotic factors such as genotype, physiological status, pathobiology, the lifestyle of the hosts, and the use of probiotics, as well environmental factors, which are among the most important key determinants of fecal microbiota in many aquatic species, including silver carp, bighead carp, and common carp (Yukgehnaish et al., 2020). The correlation between the environment and the components of the gut microbiota might be caused by multiple factors (Yukgehnaish et al., 2020), such as the availability of wild plant materials (Eichmiller et al., 2016) and the physiochemical status of rearing water (Giatsis et al., 2015). Regarding red swamp crayfish cultured in ponds (Zhang Z. et al., 2020) and rice paddy fields (Shui et al., 2020), the research on gut microbiota patterns mainly focused on microbial composition based on high-throughput sequencing of 16S rRNA genes. Among these, there have been few functional studies, which have also been based on 16S rRNA gene sequences. Compared with high-throughput sequencing of 16S rRNA genes, metagenomics can be used not only to reflect the complexity of microbial communities but also to uncover the functional capacity of the gut microbiome (Xing et al., 2014). Furthermore, Kyoto Encyclopedia of Genes and Genomes (KEGG) and Carbohydrate-Active enZYmes (CAZy) databases based on metagenomics can provide systemic functional information related to metabolism, human diseases, etc. This information can help to understand aspects such as the metabolic characteristics of crayfish and to identify genes of microorganisms potentially pathogenic to human beings.

In the present study, our aim was to investigate the functional diversities of the gut microbiota from red swamp crayfish cultured in aquaculture pond and rice paddy field environments. To that aim, we performed a metagenomics analysis based on the NR, KEGG, and CAZy databases. We assumed that the microbial community composition, some of the microbial functions in the KEGG pathway, and genomic, structural, and biochemical information on carbohydrate-active enzymes (CAZymes) in the intestines of crayfish would vary between the two different culture environments due to differences in nutrient input and ecological community composition. Our findings will be helpful in assessing the effects of the external environment on the structure, functions, and mechanisms of the crayfish gut microbial community.

## Materials and Methods

### Sample Collection

Eight ponds and six rice-crayfish cultivation fields were used in this study. Four earthen ponds were located at the Xuyi Scientific Research and Breeding Farm of the Freshwater Fisheries Research Center, Chinese Academy of Fishery Sciences (32.994214°, 118.672861°), and the other four ponds were located in the Green Valley Eco-Farm rice-crayfish cultivation fields in Xuyi County, Jiangsu Province (33.043494°, 118.681004°). All six rice-crayfish fields were located at the Green Valley Eco farm. All the samples were collected on July 11, 2019. The pond conditions and cultivation process of all red swamp crayfish aquaculture ponds in these two neighboring farms were similar. Each pond had an area of approximately 0.5–0.66 ha. Elodea (*Elodea nuttallii*) was planted in early March 2019 in approximately 40% of the surface area of the ponds. From the end of March to early April, approximately 85,000 fry/ha of red swamp crayfish (200–400/kg) were released. Six rice-crayfish cultivation fields were used to collect samples for rice-crayfish cultivation research, each with an area of approximately 0.66 ha. Each rice-crayfish cultivation field had a circular trench around it, with a depth of approximately 1 m, covering about 5% of the total area. Elodea (*E. nuttallii*) was planted in early March 2019 in approximately 40% of the surface area of the circular trench. Rice was planted in mid-June 2019, while the rice harvest time in the previous year was mid-November. After harvest, approximately 150 kg/ha of red swamp crayfish larvae (approximately 100 pieces/kg) was placed into the fields. During the period from mid-November 2018 to February 2019, the feed rate was approximately 1% and then increased to approximately 3% of the body weight. A feed specially formulated for *P. clarkii* was provided (crude protein ≥26%, crude fiber ≤18%, crude ash ≤15%, calcium 0.3–0.4%, total phosphorus ≥0.4%, crude fat ≥3%, water ≤13%, and lysine ≥1%). During the whole breeding period, rice special compound fertilizer (total nutrient ≥25%, N13-P6-K6) was used at about 525 kg/ha. Finally, the crayfish yield was approximately 3,500 and 1,500 kg/ha in aquaculture ponds and rice-crayfish cultivation fields, respectively.

Five 1-year-old adult crayfish were randomly collected from each field and each pond at the end of the cultivation, and the whole intestines were sampled to make five replicates of each field and pond. The samples from the six fields were marked as GCRF1–6, while the samples from the eight ponds were marked as GCF1–8. After separation and collection, the contents in the intestines were placed into 2-ml sterilized plastic sample storage tubes, transported to the laboratory with liquid nitrogen, and then stored at −80°C immediately before DNA extraction.

### Metagenomics Analysis: DNA Extraction, Illumina Sequencing, and Assembly

Genomic DNA was extracted from 200 mg of intestinal contents using a QiAamp DNA stool Mini Kit (Qiagen, Valencia, CA, United States) according to the manufacturer’s protocol. The extracted DNA was quantified using a Qubit dsDNA HS Assay Kit (Invitrogen, Carlsbad, CA, United States). Equal amounts of DNA from five replicates of the same pond or field were equivalently mixed to make the final samples of each pond or field. A Covaris S220 ultrasonic DNA breaker (Covaris, Woburn, MA, United States) was used for DNA fragmentation. The NEB Next^®^ Ultra^TM^ DNA Library Prep Kit for Illumina^®^ (NEB, Ipswich, MA, United States) and T100^TM^ Thermal Cycler PCR instrument (Bio-Rad, Hercules, CA, United States) were used to construct the DNA library. The Illumina HiSeq 2500 sequencing platform and HiSeq paired-end cluster generation kit (Illumina, San Diego, CA, United States) were used for high-throughput sequencing.

Fastqc software was used to evaluate the quality of the sequencing data. Trimmomatic software was used to filter the sequencing data. IDBA_UD software was used for splice and assembly of high-quality reads. The contigs were obtained based on overlaps between reads. The best KMER assembly results were selected through a comprehensive evaluation of the assembly results of several KMERs. Prokaryotic dynamic programming genetic algorithm (Prodigal) was used to predict the open reading frame (ORF) of the splicing results, and the genes with lengths greater than or equal to 100 bp were selected and translated into the amino acid sequence. CD-HIT software was used to remove the redundancy of the gene prediction results of each sample to obtain the non-redundant gene set. The gene set was compared with the NR, KEGG, and CAZy databases to obtain species annotation information and functional annotation information of genes. Functional abundance and species richness were determined based on the gene abundance.

### Accession Number

Raw sequence data of all samples in this study are available in the Sequence Read Archive database of National Center for Biotechnology Information (NCBI) under the accession number PRJNA671745.

### Statistical Analysis

One-way analysis of variance (ANOVA) and multiple comparisons using the Bonferroni method were performed using the SPSS 20.0. Statistical significance was set at *p* < 0.05.

## Results

Quality control was performed according to the method described above. The quality control results (Supplementary Table 1) of each sample sequence were good, indicating that the data could be used for subsequent analysis.

### The Gut Microbiota of Red Swamp Crayfish in the Two Culture Models

**FIGURE 1:**
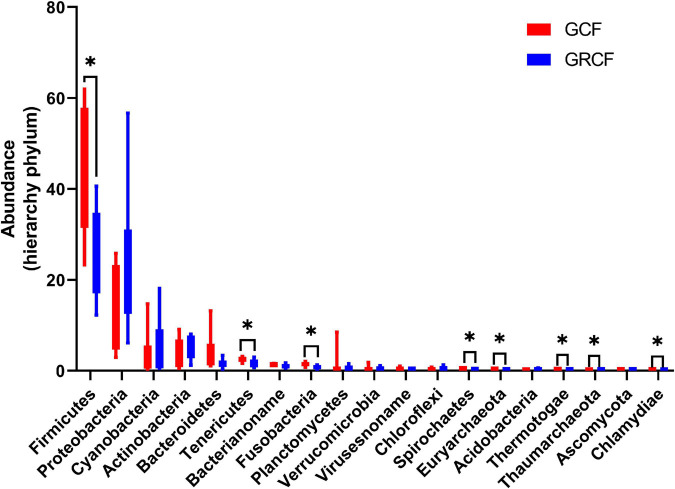
Red swamp crayfish gut taxonomic structure at the phylum level [^∗^ represents that the difference is significant (*p* < 0.05)].

Figure 1 shows the abundant phyla in the gut microbiota of red swamp crayfish. The relative abundance of bacteria was over 98% of the total; and Firmicutes, Proteobacteria, Cyanobacteria, Actinobacteria, and Bacteroidetes were the dominant phyla. Firmicutes predominated in the GCF samples (47.05% ± 14.24%), while the abundance of this phylum in GCRF samples (26.17% ± 9.28%) was significantly lower (*p* < 0.05). Other abundant phyla whose abundance significantly differed between the two groups (*p* < 0.05) were Tenericutes, Fusobacteria, Spirochaetes, Euryarchaeota (archaea), Thermotogae, Thaumarchaeota (archaea), and Chlamydiae.

The dominant archaeal phyla in GCF samples were Euryarchaeota (79.40% ± 8.19%), Thaumarchaeota (9.23% ± 7.09%), Archaea_noname (8.73% ± 2.78%), and Crenarchaeota (1.21% ± 0.96%). The abundances of these phyla in GCRF samples were 49.75% ± 14.83%, 40.98% ± 14.52%, 3.78% ± 1.45%, and 3.79% ± 1.35% for Euryarchaeota, Thaumarchaeota, Archaea_noname, and Crenarchaeota, respectively, which were significantly different from those in GCF samples (*p* < 0.05) (Supplementary Figure 1A). The dominant fungal phyla were Ascomycota and Basidiomycota (Supplementary Figure 1B); and the dominant viral families were *Virusesnoname*, *Siphoviridae*, *Podoviridae*, *Myoviridae*, *Herpesviridae*, *Mimiviridae*, and *Polydnaviridae* (Supplementary Figure 1C). No significant differences in the abundances of fungal phyla and viral families were found between the two groups (*p* > 0.05).

In our previous study, methanogenic archaea covered the largest proportion of archaeal communities in the sediment of aquaculture ponds (Fan et al., 2018). Thus, we determined the ratio of the seven methanogenic archaeal orders, Methanobacteriales, Methanosarcinales, Methanomicrobiales, Methanomassiliicoccales, Methanococcales, Methanocellales, and Methanopyrales, to the relative abundance of archaea and their distributions in the gut of red swamp crayfish from each culture environment. The results showed that the relative abundances of these seven orders covered 62.9 and 34.77% of the total archaeal communities in the GCF and GCRF groups, respectively (Supplementary Figure 2). In the GCRF group, the relative abundance of methanogenic archaea was significantly lower (*p* < 0.05), with Methanobacteriales, Methanomicrobiales, and Methanomassiliicoccales in decreasing order of abundance.

### Kyoto Encyclopedia of Genes and Genomes Pathway Analysis

**FIGURE 2:**
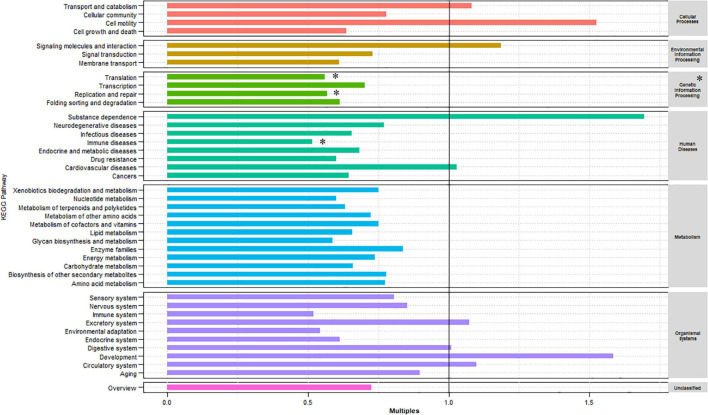
Kyoto Encyclopedia of Genes and Genomes (KEGG) categories enriched or depleted in the GCF versus GCRF components of the gut microbiome in level 1 (right) and 2 (left) [^∗^ represents that the difference is significant (*p* < 0.05)].

The KEGG database was used to examine the function of the microorganisms in the gut of red swamp crayfish from the two culture environments. Supplementary Figure 3B shows that the most abundant KEGG level 1 categories (in descending order) were metabolism, genetic information processing, and environmental information processing. Furthermore, the most abundant level 2 categories included carbohydrate metabolism, overall, amino acid metabolism, membrane transport, translation, energy metabolism, and metabolism of cofactors and vitamins (Supplementary Figure 3A). The relative abundance of the KEGG level 1 and 2 categories was compared between the two culture environments (Figure 2). Compared with the GCF group, the relative abundances of genetic information processing (level 1) as well as translation, replication and repair, and immune diseases (level 2) were significantly lower in the GCRF group (*p* < 0.05).

**FIGURE 3:**
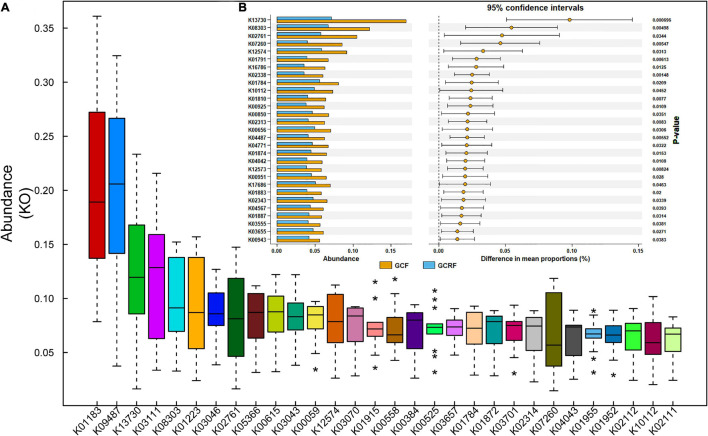
The relative abundance of abundant genes **(A)** and the genes with significant differences between the GCF and GCRF groups **(B)**. * above and below the boxplot represent the outliers.

Figure 3A shows the top 30 most abundant gene sets [genes grouped by KEGG Orthology (KO) identifiers] in all samples. The results showed that K01183 (chitinase), K09487 (HSP90B, TRA1), K13730 (bacterial invasion of epithelial cells), K03111 (single-strand DNA-binding protein), and K08303 (epithelial cell signaling in *Helicobacter pylori* infection) were the most abundant gene sets. Then, we compared the samples from the two cultivation environments in terms of the top 100 most abundant gene sets and listed those whose abundance significantly differed (*p* < 0.05) between the two cultivation environments (Figure 3B). Among them, K13730, K08303, K07260 (vancomycin resistance), and K04771 (cationic antimicrobial peptide (CAMP) resistance) are related to human diseases, while K12574, K01874, K01883, K02343, K04567, K01887, K04487, and K3655 are related to genetic information processing. In addition, there were no significant differences in the relative abundance of genes related to metabolism at both level 1 and 2 between the two environments. However, we found several differentially expressed genes related to metabolism that were grouped in K02761 (starch and sucrose metabolism), K07260 (peptidoglycan biosynthesis), K01791 (amino sugar and nucleotide sugar metabolism), and K01784 (galactose metabolism and amino sugar and nucleotide sugar metabolism). Other genes were grouped in K01810 (glycolysis/gluconeogenesis, pentose phosphate pathway, starch and sucrose metabolism, and amino sugar and nucleotide sugar metabolism), K00925 (taurine and hypotaurine metabolism, pyruvate metabolism, fructose and mannose metabolism, galactose metabolism, and methane metabolism), K00850 (glycolysis/gluconeogenesis, pentose phosphate pathway, fructose and mannose metabolism, and galactose metabolism), K00656 (pyruvate metabolism, propanoate metabolism, and butanoate metabolism), K04487 (thiamine metabolism), K04042 (amino sugar and nucleotide sugar metabolism), and K00951 (purine metabolism). Overall, most of them belonged to carbohydrate metabolism.

K13730 and K08303 were the top two most differentially abundant gene sets, and their abundances ranked third and fifth among all KO identifiers identified in the samples. K13730 and K08303 are related to the invasion of epithelial cells by *Listeria monocytogenes* and *H. pylori*, respectively. Specifically, K13730 is related to epithelial cell invasion by *Listeria* through InlA (PizarroCerdá et al., 2002). The abundance of *L. monocytogenes* in GCF and GCRF samples was compared (Supplementary Figure 4A), and correlation analysis was performed (Supplementary Figure 4B) between the abundance of *L. monocytogenes* and that of K13730. The results showed that the abundance of *L. monocytogenes* was higher in GCF than in GCRF samples, although the difference was not significant (*p* > 0.05). Moreover, the abundance of *L. monocytogenes* was significantly and positively correlated with that of K13730 (*p* < 0.05). K08303 is related to epithelial cell invasion by *H. pylori* during mucus digestion. The abundance of *H. pylori* in GCF and GCRF samples was also compared (Supplementary Figure 5A), and correlation analysis was also performed between the abundance of *H. pylori*, the abundance of *Helicobacter* genus, and that of K08303 (Supplementary Figures 5B,C). The results showed that the abundance of *H. pylori* was higher in GCF than in GCRF samples (*p* < 0.05). Moreover, the abundance of the *Helicobacter* genus, but not of *H. pylori*, was significantly and positively correlated with that of K08303 (*p* < 0.05).

### Carbohydrate-Active Enzyme Analysis and the Effects of Rice Cultivation

**FIGURE 4:**
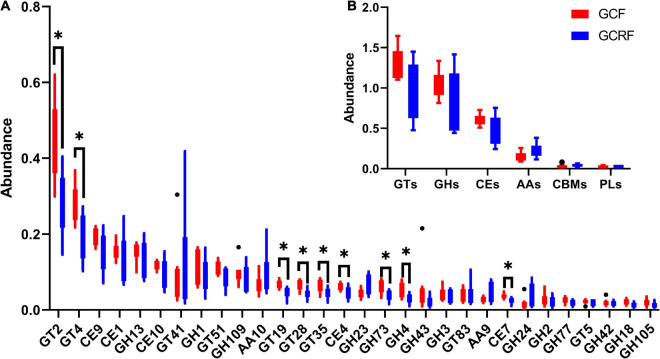
Red swamp crayfish gut carbohydrate-active enzymes (CAZymes) **(B)** and the subfamilies **(A)** [^∗^ represents the difference is significant (*p* < 0.05)]. The black dots above and below the boxplot represent the outliers.

Carbohydrate-active enzymes was used to investigate glycoside hydrolases (GHs), polysaccharide lyases (PLs), carbohydrate esterases (CEs), glycosyltransferases (GTs), auxiliary activities (AAs), and carbohydrate-binding modules (CBMs) based on sequence similarity (Lombard et al., 2014). The results are presented in Figure 4B. GTs and GHs were the two most abundant categories. The abundances of GTs, GHs, CEs, AAs, CBMs, and PLs decreased gradually in both cultivation environments. Moreover, the ratio of GHs/GTs was generally higher in the GCRF group than in the GCF group, although no significant differences were found (*p* > 0.05). Further research on the subfamilies based on sequence similarity showed that GT2 was the most abundant functional group (Figure 4A). Furthermore, GT2 was significantly more abundant in the GCF group than in the GCRF group (*p* < 0.05). Some other subfamilies whose abundance was significantly higher in the GCF group included GT4, GT19, GT28, GT35, CE4, and CE7.

## Discussion

The red swamp crayfish, *P. clarkii*, is an important farmed aquatic species in China that is grown in aquaculture ponds and rice paddy fields. These are two distinct cultivation environments; therefore, they may have different effects on the growth and/or physiological status of crayfish. The gut microbiota, which is greatly influenced by the environment, plays a critical role in host development, physiology, and health. Thus, the effects of the external environment on the structure, functions, and mechanisms of the crayfish gut microbial community should be understood. We hypothesized that the microbial community composition and functions would vary between the two different culture environments due to differences in nutrient input and ecological community composition. Here, we sought to examine this hypothesis by investigating the functional diversities of the gut microbiota from red swamp crayfish cultured in aquaculture ponds (GCF group) and rice paddy fields (GCRF group). To that aim, we performed a metagenomics analysis based on the NR, KEGG, and CAZy databases.

In pursuing the aims mentioned above, we found that (1) there were significant differences in the relative abundance of the dominant bacterial phyla and archaeal orders between the two groups, with the GCRF group showing significantly lower relative abundance of Firmicutes and methanogenic archaea; (2) the relative abundances of the KEGG level 1 category, genetic information processing, and the level 2 categories, translation, replication and repair, and immune diseases were significantly lower in the GCF group; (3) K13730 and K08303, which are related to epithelial cell invasion by *L. monocytogenes* and *H. pylori*, respectively, were the top two most differentially abundant KO gene sets, and that the abundances of *L. monocytogenes* and *H. pylori* were higher in the GCF group than in the GCRF group; and (4) GTs and GHs were the two most abundant categories among the CAZymes families, with a higher (not significant) ratio of GHs/GTs and a significantly lower abundance of the subfamilies GT2, GT4, GT19, GT28, GT35, CE4, and CE7 in the GCRF group than in the GCF group.

### Differences in the Gut Microbiota Composition Between the Two Culture Environments

Other researchers have previously reported that the dominant bacterial phyla in the intestines of red swamp crayfish, from both aquaculture ponds (Zhang Z. et al., 2020) and rice paddy fields (Shui et al., 2020), are Actinobacteria, Proteobacteria, Tenericutes, Firmicutes, and Bacteroidetes. Similarly, we also found these five phyla among the six most abundant phyla. Nevertheless, we found that Cyanobacteria was also one of the dominant phyla. This might indicate that Cyanobacteria in the environment can enter the intestines of red swamp crayfish and develop into one of the dominant compositions. It has been reported that red swamp crayfish can promote the release of Cyanobacteria from the sediment by bioturbation (Yamamoto, 2010) and influence the phytoplankton community, including Cyanobacteria (Gherardi, 2006) in the water. Thus, Cyanobacteria may become one of the dominant bacterial community compositions in the intestines of crayfish independently from whether crayfish can digest it or not.

The bacterial and archaeal, but not fungal and viral, community compositions presented some significant differences between the two groups. Firmicutes, Proteobacteria, Actinobacteria, Bacteroidetes, Tenericutes, and Fusobacteria have been shown to be the most abundant bacterial phyla in the gut of red swamp crayfish (Shui et al., 2020; Ye et al., 2020; Zhang Y. et al., 2020; Zhang Z. et al., 2020). Many external factors, such as heavy metals (Zhang Y. et al., 2020), dietary and geographical conditions (Zhang Z. et al., 2020), and bacterial community compositions in sediment (Ye et al., 2020), can affect the relative abundances of these abundant phyla in the guts of red swamp crayfish. Even the different fertilization modes in rice-crayfish cultivation environments can also influence their relative abundances (Huang, 2019). Thus, the fact that the relative abundances of Firmicutes and Proteobacteria were significantly lower and higher, respectively, in the GCRF group than in the GCF group might be related to factors that differed between these two culture models. Among the factors, the planting of rice may be especially important, as different crayfish-plant co-culture ecosystems have been shown to result in distinct bacterial communities in the intestines of crayfish (Wei et al., 2021). The ecological interactions between the microbial communities in the intestines of crayfish and the environments cause variations in the gut microbiota composition between these two culture environments (Wang et al., 2021). Nevertheless, bacterial communities that perform major functions in the guts of red swamp crayfish can change significantly, even at the phylum level. In addition, the compositions of the abundant bacterial phyla were relatively stable; however, their relative abundances changed; and Firmicutes, Proteobacteria, and Actinobacteria could all be the most abundant phyla.

The ratio of archaeal community compositions to the total was less than 1%; however, there were differences between the two culture models. Among the seven methanogenic orders, Methanobacteriales, Methanomicrobiales, and Methanomassiliicoccales presented significantly lower relative abundances in the GCRF group. Thus, we can consider that methane production by these three orders was potentially lower in the guts of crayfish from this group. To date, there have been no reports on methane production in the intestines of crayfish; however, the existence of methanogens and the anaerobic environment that the gut can provide made us consider the possibility of methane production in the guts of crayfish. Methane production has been detected in the feces of nearly all herbivorous and some omnivorous and carnivorous terrestrial vertebrates (Franz et al., 2011). Meanwhile, the assumption that small herbivores produce negligible amounts of methane (Hackstein and van Alen, 1996) does not consider significant changes in their biomass. In addition, the relative abundance of methanogens covered more than 60% of the total archaeal composition in the guts of red swamp crayfish from culture ponds, which might indicate that they presented most of the archaeal functions. Given the process of bioturbation, the effects on methanogenic community compositions and the variations in their relative abundances between the two groups were attributed to differences in the water and sediment environments. These may include different methanogenic community compositions, different physical and chemical properties, and/or different food compositions brought by the different environments. Further research is needed to address this issue.

### Differences in the Function of the Gut Microbiota Between the Two Culture Models

Kyoto Encyclopedia of Genes and Genomes pathway analysis revealed that the abundance of genes involved in pathways primarily involved in genetic information processing and human diseases at level 1, and translation, replication and repair, and immune disease in level 2 was lower in GCRF samples. This might indicate that the function of genetic information processing, the community composition in the gut of red swamp crayfish, was more sensitive to variations in the culture environment in the present study. The cultivation of rice and the consequent changes in food composition caused by the complexity of the ecosystem reduced the use of compound feed, which may have affected the proliferation of microorganisms in the digestive tract of red swamp crayfish. However, the changes were not sufficiently drastic to cause significant changes in most of the functions.

The genes associated with human diseases might be related to human activities in this area. K13730 and K08303, the top two most differentially abundant KO gene sets in the two culture models, were related to the invasion of epithelial cells by *L. monocytogenes* and *H. pylori*, respectively. The abundance of *L. monocytogenes* was significantly and positively correlated with that of K13730, indicating that the K13730 group might originate mainly from *L. monocytogenes* in the gut of red swamp crayfish. In contrast, the results showed that the abundance of *Helicobacter* genus, not that of *H. pylori*, was significantly and positively correlated with that of K08303, indicating that the K08303 group may originate not only from *H. pylori*, but also from other bacterial strains, especially in the *Helicobacter* genus. This may have helped to digest gastric mucus, thus facilitating the invasion of gastric epithelial cells by *Helicobacter* sp. *L. monocytogenes* was reported to induce dysbiosis in snails (Dushku et al., 2020), and *H. pylori* is thought to be a new fish-borne pathogen of tilapia (Abdel-Moein et al., 2015). Thus, *L. monocytogenes* and *H. pylori* may attack the gut of crayfish. Attention should be paid to avoid the entry of untreated human feces into the aquaculture environment. Importantly, some pathogens in ambient culture environments were reported to be capable of colonizing the intestines of crayfish (Sinnott, 1990).

Crayfish from the rice-crayfish cultivation environments had lower abundances of *L. monocytogenes* and *H. pylori* in their intestines than those from monoculture environments. This indicated that there were factors in rice-crayfish cultivation environments that reduced the abundance of *L. monocytogenes* and *H. pylori* in the intestines of crayfish. Thus, the rice-crayfish cultivation environment reduced the risk of colonization by these two potential pathogens and reduced the potential risk to human beings. This needs to be further investigated.

In addition, although KEGG pathway analysis did not reveal differences in genes related to metabolism in levels 1 and 2 between the two culture environments, CAZymes analysis revealed significant differences of abundance in subfamilies of these enzymes, including GT2, GT4, GT19, GT28, GT35, CE4, and CE7. This indicated that there existed factors influencing the metabolism of, at least, these subfamilies of carbohydrates. GTs constituted the most abundant category among the six functional families, followed by GHs, which indicated that the biosynthesis of functional disaccharides, oligosaccharides, and polysaccharides was the most important carbohydrate metabolic feature (Feng et al., 2021), especially in the GCF group. This is different from the carbohydrate metabolic features in the rumen of two kinds of herbivorous animals, Yak (Yajing et al., 2019) and Haizi Buffalo (Huimin et al., 2017), in which GHs constitute the most abundant family of CAZymes for hydrolysis and rearrangement of glycosidic bonds (Davies and Henrissat, 1995). In addition, the relative abundance of GHs tended to be higher in GCRF samples, which indicated that the red swamp crayfish in the GCRF group might also feed on some plant foods. GT2, which was the most abundant subfamily of CAZymes in the gut of red swamp crayfish, exhibited significantly higher relative abundance in the GCF group than in the GCRF group. This subfamily includes synthases for bacterial and embryophyte cellulose, invertebrate chitin and chitooligosaccharides, and bacterial and vertebrate hyaluronan (Stanisich and Stone, 2009). We speculate that in this case, it was related to bacterial cellulose synthesis, which is a key component of the extracellular matrix that coats the cells to form cell aggregates (Ross, 1991) and biofilms (Galperin and Shalaeva, 2018) in the gut of red swamp crayfish. Thus, it is likely that the microorganisms in the guts of red swamp crayfish from the GCF group had a stronger biofilm-forming ability and that some factors in rice-crayfish environments reduced this kind of ability. This also indicated that the treatment of red swamp crayfish with intestinal diseases might be improved after placing them into a rice-crayfish environment because pathogenic bacteria may be protected in a stable biofilm.

## Conclusion

In summary, we found that the gut microbiota of red swamp crayfish grown in pond culture and rice-crayfish cultivation environments differs in composition and function. In terms of microbial community composition, there were significant differences in bacterial and archaeal community composition between the two groups. The abundance of Firmicutes and methanogenic archaea, especially the orders of Methanobacteriales, Methanomicrobiales, and Methanomassiliicoccales, was lower, while that of Proteobacteria was higher in the guts of crayfish grown in rice-crayfish cultivation environments than in crayfish grown in aquaculture ponds. Moreover, rice-crayfish cultivation resulted in a reduction in the abundance of genes involved in pathways related to genetic information processing and human diseases. Furthermore, the abundance of the K13730 and K08303 gene sets, which consist of genes related to epithelial cell invasion by *L. monocytogenes* and *H. pylori*, respectively, was lower in the guts of crayfish grown in rice-crayfish cultivation environments. GTs were the most abundant CAZymes, and GT2 was the most abundant subfamily, in both groups. However, the gut microbiota of crayfish grown in rice-crayfish cultivation environments tended to have lower abundance of GTs, including GT2, and higher abundance of GHs than that of crayfish grown in aquaculture ponds.

## Data Availability Statement

Raw sequence data of all samples in this study are available in Sequence Read Archive database of NCBI under the SRA Accession: PRJNA671745.

## Ethics Statement

The animal study was reviewed and approved by the Ethics Committee of Freshwater Fisheries Research Center, CAFS.

## Author Contributions

LF, XC, and JC provided the ideas. SM and DL conducted the sample collection and sample preparation. LQ, XD, and QW conducted the DNA extraction and detection of water and sediment parameters. GH analyzed the preliminary data. LF finished the original draft and polished by XC. All authors read and contributed to the manuscript.

## Conflict of Interest

QW is employed by Wuxi COFCO Engineering and Technology Co., Ltd. She was once a student of Wuxi Fishery College, Nanjing Agricultural University, and participated in some of its research. The remaining authors declare that their research was conducted in the absence of any commercial or financial relationships that could be construed as a potential conflict of interest.

## Publisher’s Note

All claims expressed in this article are solely those of the authors and do not necessarily represent those of their affiliated organizations, or those of the publisher, the editors and the reviewers. Any product that may be evaluated in this article, or claim that may be made by its manufacturer, is not guaranteed or endorsed by the publisher.

## Footnotes

1
http://www.moa.gov.cn/
