# Supplementary material for: Metagenomics Analysis Reveals Compositional and Functional Differences in the Gut Microbiota of Red Swamp Crayfish, Procambarus clarkii, Grown on Two Different Culture Environments
Source: Front Microbiol. 2021 Oct 18;12:735190. doi: 10.3389/fmicb.2021.735190 (PMC8558459; doi:10.3389/fmicb.2021.735190)
Supplement: Supplementary file 1 [file Data_Sheet_1.docx]

Supplementary Material

# Supplementary Figures and Tables

## Supplementary Figures


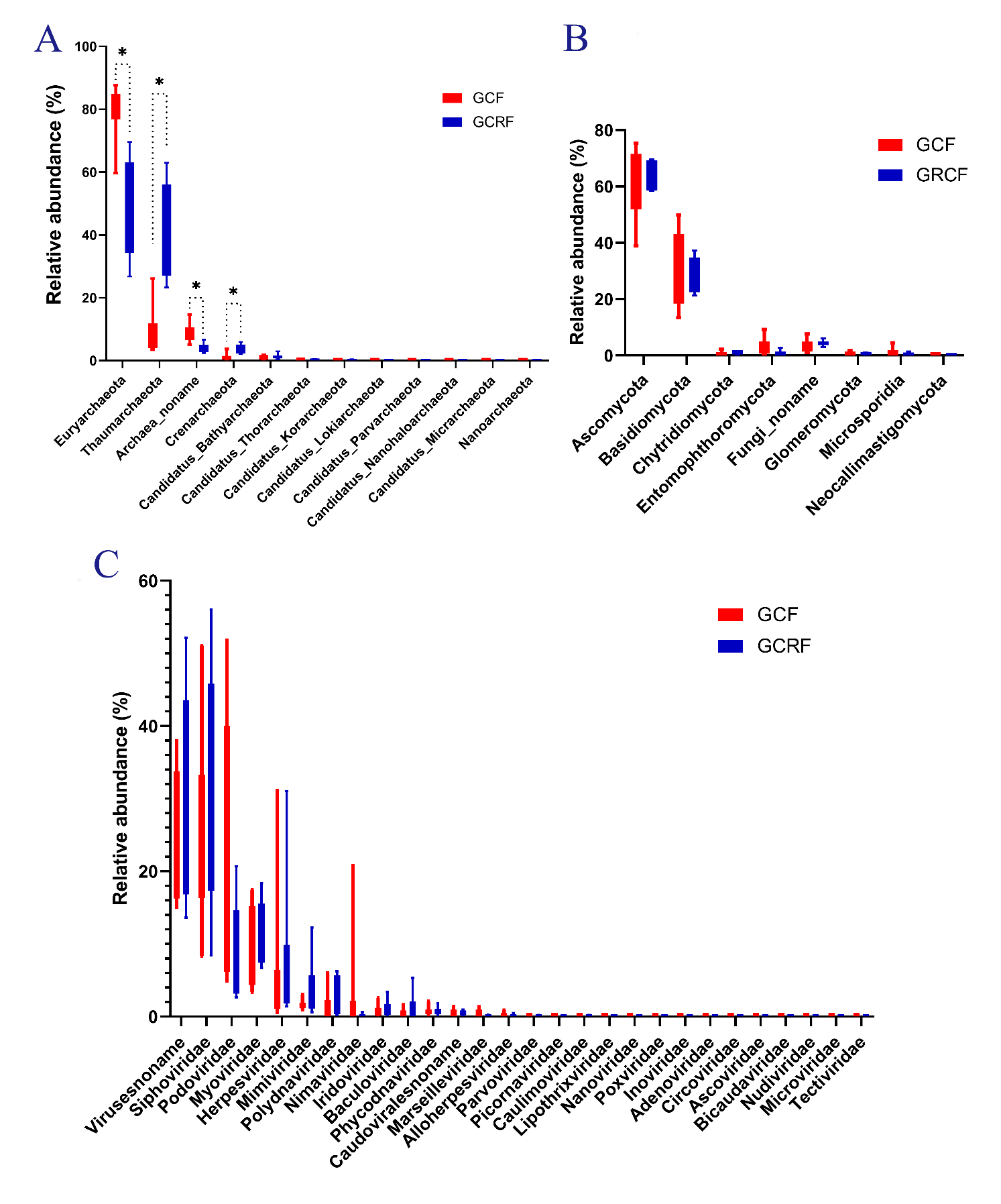


**Supplementary Figure 1.** Red swamp crayfish gut taxonomic structure (A, B, C represent the abundant archaeal phyla, fungal phyla, and the viral families respectively. And *represents the difference is significant (p ＜0.05)).

**
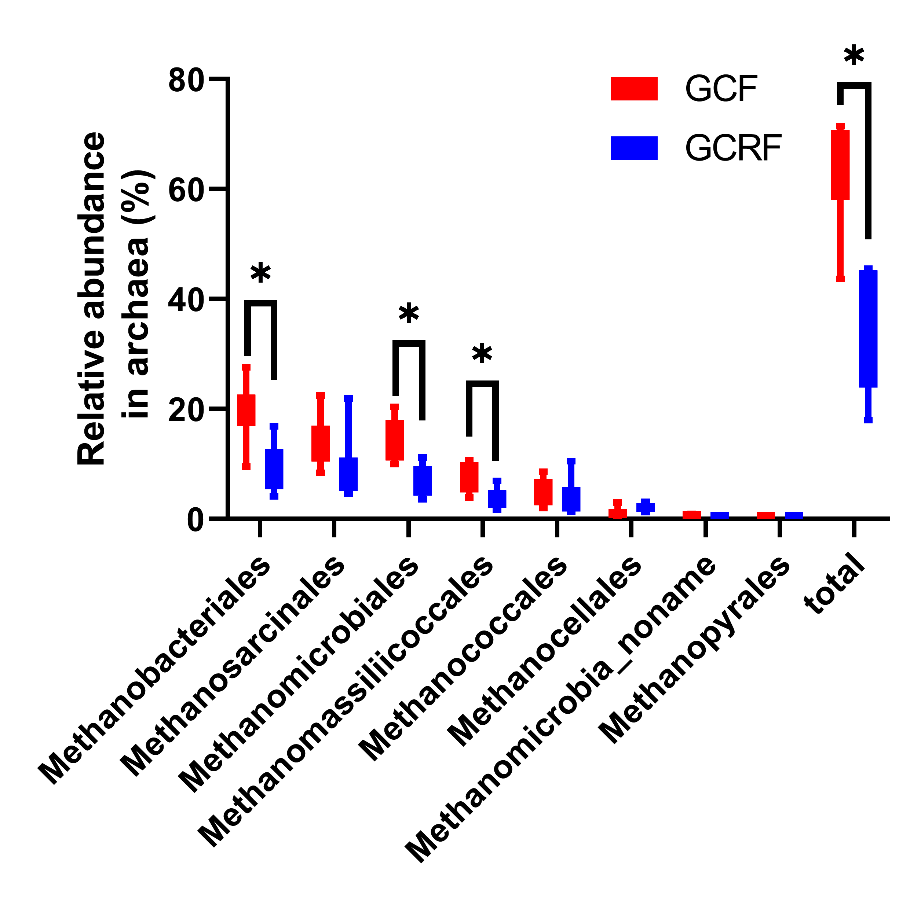
**

**Supplementary Figure 2.** Methanogenic archaeal orders in the gut of red swamp crayfish. (*represents the difference is significant (p ＜0.05)).

.


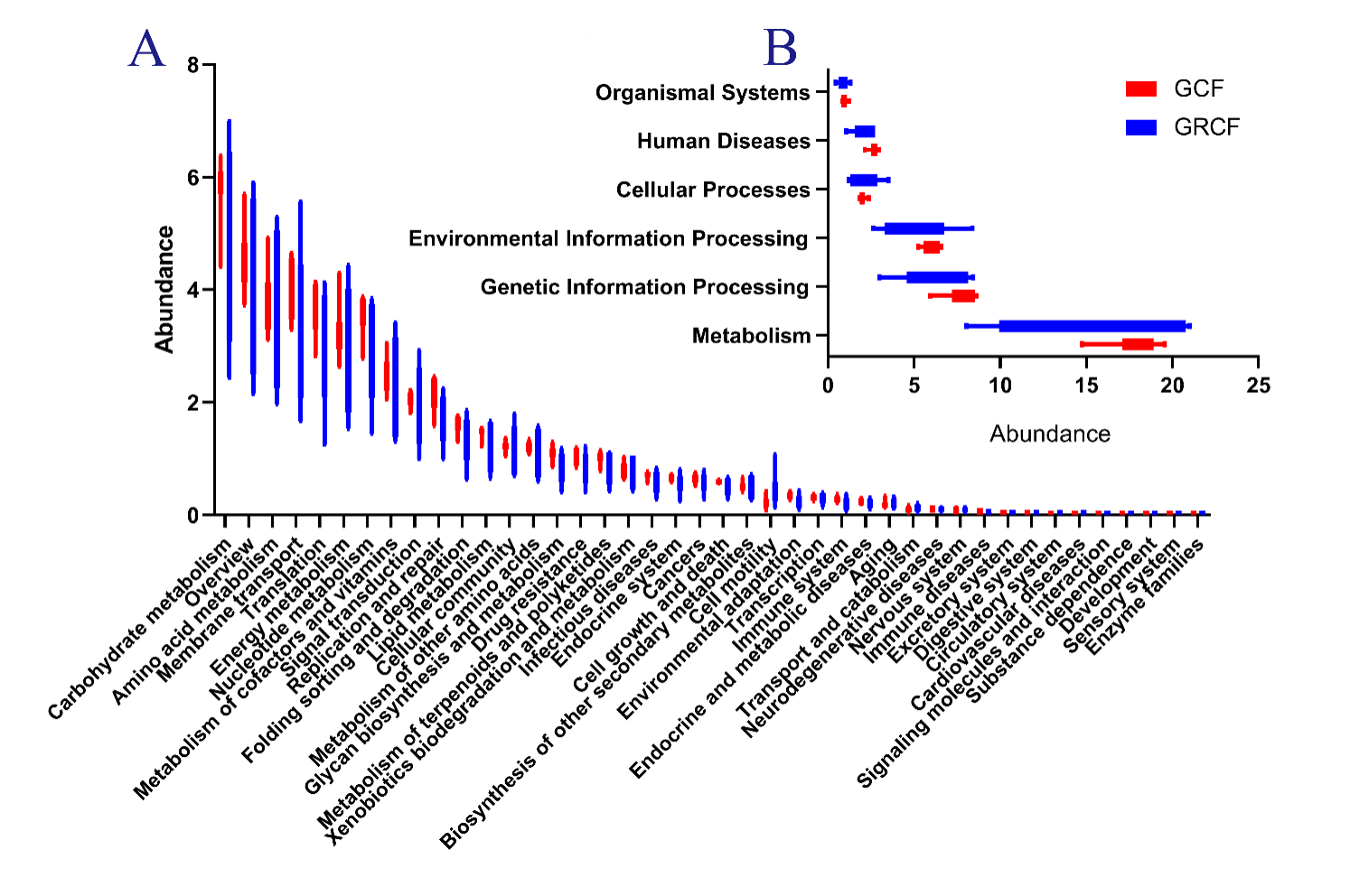


**Supplementary Figure 3.** KEGG pathway classifications in Level1(A) and Level2 (B).


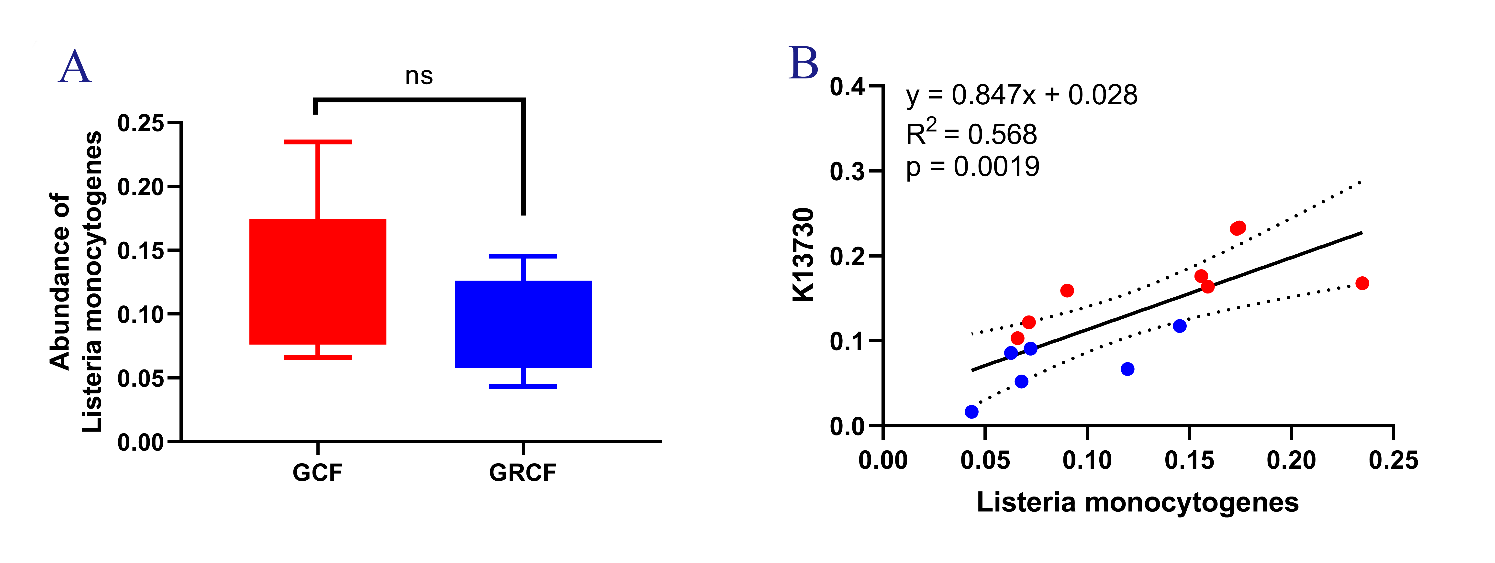


**Supplementary Figure 4.** Abundance of *Listeria monocytogenes* in the gut of red swamp crayfish (A) and correlation analysis with the abundance of the gene set, K13730 (B). (ns means that there is no significant differences between these two groups (p ＞0.05))


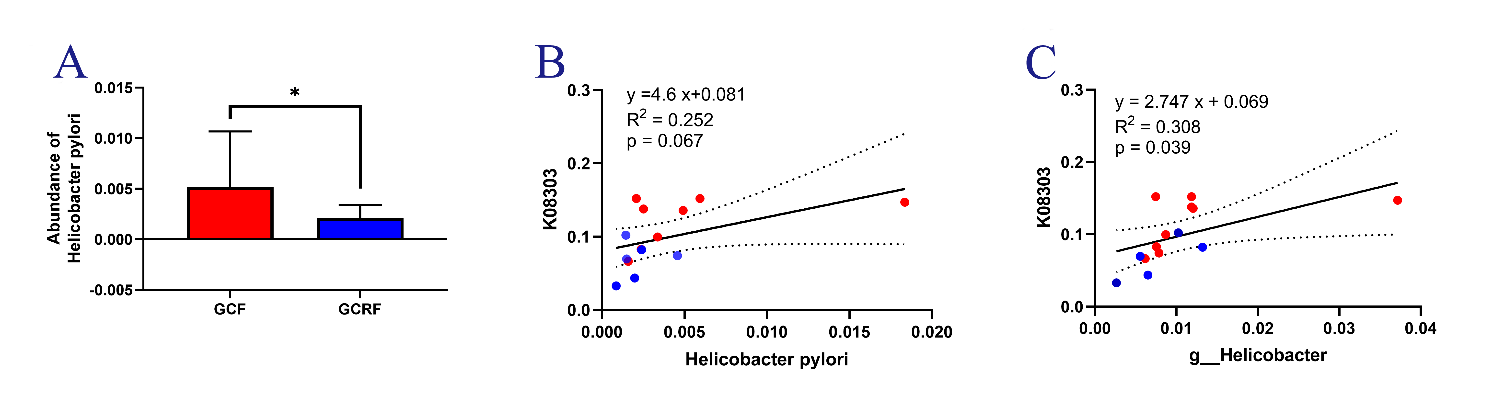


**Supplementary Figure 5.**Abundance of *Helicobacter pylori* in the gut of red swamp crayfish (A) ， correlation analysis with the abundance of the gene set, K08303 (B)and correlation analysis on the abundance of *Helicobacter* genus with the abundance of gene set, K08303.(*represents the difference is significant (p ＜0.05)).

|  | GRCF1 | GRCF2 | GRCF3 | GRCF4 | GRCF5 | GRCF6 | GCF1 | GCF2 | GCF3 | GCF4 | GCF5 | GCF6 | GCF7 | GCF8 |
| --- | --- | --- | --- | --- | --- | --- | --- | --- | --- | --- | --- | --- | --- | --- |
| Total Reads Count(#) | 36392934 | 41603618 | 42214782 | 38318352 | 41815912 | 43747492 | 46514278 | 37747112 | 37269930 | 40897480 | 38972026 | 44205232 | 44587790 | 41422292 |
| Total Bases Count(bp) | 5.37E+09 | 6.09E+09 | 6.22E+09 | 5.56E+09 | 6.04E+09 | 6.35E+09 | 6.77E+09 | 5.49E+09 | 5.4E+09 | 6E+09 | 5.71E+09 | 6.46E+09 | 6.51E+09 | 6.07E+09 |
| Average Read Length(bp) | 147.45 | 146.3 | 147.24 | 145.13 | 144.55 | 145.19 | 145.64 | 145.47 | 144.94 | 146.62 | 146.59 | 146.06 | 146.1 | 146.51 |
| Q10 Bases Count(bp) | 5.37E+09 | 6.09E+09 | 6.22E+09 | 5.56E+09 | 6.04E+09 | 6.35E+09 | 6.77E+09 | 5.49E+09 | 5.4E+09 | 6E+09 | 5.71E+09 | 6.46E+09 | 6.51E+09 | 6.07E+09 |
| Q10 Bases Ratio(%) | 100.00% | 100.00% | 100.00% | 100.00% | 100.00% | 100.00% | 100.00% | 100.00% | 100.00% | 100.00% | 100.00% | 100.00% | 100.00% | 100.00% |
| Q20 Bases Count(bp) | 5.29E+09 | 5.99E+09 | 6.12E+09 | 5.47E+09 | 5.95E+09 | 6.26E+09 | 6.67E+09 | 5.41E+09 | 5.32E+09 | 5.91E+09 | 5.63E+09 | 6.36E+09 | 6.42E+09 | 5.97E+09 |
| Q20 Bases Ratio(%) | 98.56% | 98.39% | 98.44% | 98.38% | 98.37% | 98.51% | 98.42% | 98.51% | 98.44% | 98.48% | 98.51% | 98.52% | 98.57% | 98.43% |
| Q30 Bases Count(bp) | 5.08E+09 | 5.74E+09 | 5.87E+09 | 5.24E+09 | 5.69E+09 | 6.02E+09 | 6.38E+09 | 5.17E+09 | 5.08E+09 | 5.67E+09 | 5.41E+09 | 6.12E+09 | 6.19E+09 | 5.73E+09 |
| Q30 Bases Ratio(%) | 94.66% | 94.30% | 94.38% | 94.18% | 94.09% | 94.81% | 94.12% | 94.22% | 94.03% | 94.53% | 94.76% | 94.76% | 94.97% | 94.40% |
| N Bases Count(bp) | 731 | 25490 | 24432 | 777 | 14573 | 3867 | 19553 | 695 | 702 | 3986 | 3882 | 3902 | 3863 | 3995 |
| N Bases Ratio(%) | 0.00% | 0.00% | 0.00% | 0.00% | 0.00% | 0.00% | 0.00% | 0.00% | 0.00% | 0.00% | 0.00% | 0.00% | 0.00% | 0.00% |
| GC Bases Count(bp) | 2.83E+09 | 3.24E+09 | 3.16E+09 | 2.79E+09 | 2.63E+09 | 3.38E+09 | 2.62E+09 | 1.98E+09 | 1.85E+09 | 2.26E+09 | 2.71E+09 | 3E+09 | 3.46E+09 | 2.43E+09 |
| GC Bases Ratio(%) | 52.76% | 53.28% | 50.80% | 50.08% | 43.45% | 53.14% | 38.62% | 35.99% | 34.18% | 37.66% | 47.51% | 46.44% | 53.14% | 39.99% |

## SupplementaryTable

Supplementary Table.1 Raw data statistics for all samples
